# Correlation between Pacifier Use in Preterm Neonates and Breastfeeding in Infancy: A Systematic Review

**DOI:** 10.3390/children9101585

**Published:** 2022-10-19

**Authors:** Eirini Orovou, Maria Tzitiridou-Chatzopoulou, Maria Dagla, Panagiotis Eskitzis, Ermioni Palaska, Maria Iliadou, Georgios Iatrakis, Evangelia Antoniou

**Affiliations:** 1 Department of Midwifery, University of Western Macedonia, Keptse, 50200 Ptolemaida, Greece; 2 Department of Midwifery, University of West Attica, Agioy Spyridonos 28, 12243 Egaleo, Greece

**Keywords:** preterm neonate, pacifier, pacifier use, breastfeeding, NICU

## Abstract

Background: Breastfeeding is very important for the proper nutrition and growth of the child, as well as, the health of the mother. To start breastfeeding, the neonate must have extensive oral capacities for sucking functions but, premature neonates may not have the muscle strength needed to suck successfully. However, the non-nutritive sucking achieved by using a pacifier, has been identified by previous research as a factor associated with shorter duration and exclusivity of breastfeeding. This study aims to perform a systematic review to investigate the relationship between pacifier use in preterm neonates and breastfeeding in infancy. Methods: We included prospective studies, as well as randomized controlled studies that evaluated the association between pacifier use by preterm neonates and of breastfeeding in infancy. Ten research articles from PubMed/Medline, Google Scholar and Crossref were included in the review from a total of 1455 articles. The results differ depending on the type of study.Most prospective studies have shown a negative correlation between pacifier use and breastfeeding, while the randomized controlled studies found a positive correlation. Conclusions: Pacifier use in preterm infants helps transition from tube to oral feeding, breastfeeding, faster weight gain and earlier discharge from the NICU. However, the relationship between pacifiers and breastfeeding is more complicated, as it appears to be influenced by additional risk factors.

## 1. Introduction

Sucking is an important milestone for any neonate which allows exclusive breastfeeding and also contributes to mother-child bond [1]. Breastfeeding is very important for the proper nutrition and growth of the child as well as the health of the mother. The World Health Organization (WHO) recommends exclusive breastfeeding for the first 6 months of life, followed by continued breastfeeding with appropriate solid foods for up to 2 years and beyond [2]. Unfortunately, the duration of exclusive breastfeeding remains significantly lower all over the world [3,4].

To start breastfeeding, the neonate must have extensive oral capacities for sucking functions. The two patterns with which the neonate acquires these abilities are the non-nutritive sucking (NNS) and nutritive sucking (NS). NNS occurs in the absence of food supply, when the infant sucks a finger or a pacifier. NNS also, can be accomplished at a non-lactating or low-lactating nipple as well [5]. It is a precursor to nutritious sucking and has several physiological benefits such as improved digestion, behavioral organization and pain management [6,7], while NS occurs when the infant uses a baby bottle or during breastfeeding [8]. NNS is a fundamental infant skill that is important for oral feeding and self-regulation. It begins long before a neonate is born and has been observed since the 15th week of intrauterine life [9]. Of course, NNS in the neonate is a primitive reflex that is predictable [10]; however, it can be affected by a preterm birth. In particular, premature neonates may not have the muscle strength needed to suck successfully, in contrast to the full-term neonates [9]. The risk of loss of the sucking reflex has been identified in neonates who are separated from their mothers for long periods of time due toNeonatal Intensive Care Unit (NICU) hospitalization [11,12]. Stimulation of NNS through the pacifier in NICU isthe facilitator to start sucking feeding, with the aim of reducing the duration of hospital stay and the start of breastfeeding [13]. Breast milk for premature neonates represents a real opportunity for health, however, the importance of breastfeeding is unlimited in terms of the proper development of the child and the prevention of some diseases in the mother [2,14], and therefore, NNS stimulation is often recommended to be performed with the glove finger, avoiding artificial nipples, so as not to interfere with breastfeeding [15]. On the other hand, the pacifier in NICU in addition to the lower risk of Sudden Infant Death Syndrome (SIDS) is also used to relieve pain during invasive procedures performed on neonates [16,17]. In these cases the contribution of NNS with a pacifier has been emphasized in maintaining the reflex and enhancing the normal stability in infants [18].

The offer of a pacifier in order to stimulate NNS and its use by infants represents a cultural phenomenon created by the previous generations. More specifically, the use of the pacifier can be a habit that has a cultural background based on the customs of the population. As a cultural object, the pacifier is related to the social representation of a calm child and a recumbent mother [19]. However, this use has been identified as a factor linked with lower duration and exclusivity of breastfeeding [7,20].

The American Academy of Pediatrics (AAP) Section on Breastfeeding recommends avoiding pacifier exposure until breastfeeding is fully established at about 3–4 weeks of the infant’s life in order to avoid “nipple confusion” [21], that is the difficulty or preference of one infant for one feeding mechanism over another after exposure to artificial nipples [22]. Regarding the risks of pacifier use, the AAP indicated that one-piece pacifiers are less likely to break and pose a choking hazard, stressing that the shield must be stable and large enough so that it does not go completely into the mouth [23]. Pacifier use also needs attention because it is a risk factor for otitis media in infants and children [24,25]. On the other hand, the AAP also mentions some advantages of using a pacifier. In more details, apart from the protective effect on SIDS mentioned above, pacifier use has a beneficial effect in mothers who cannot breastfeed, provides pain relief in infants undergoing medical emergency procedures and also, reduces the likelihood of finger sucking habit [23]. Furthermore, the AAP also published recommendations for the use of pacifiers in healthy neonates associated with a reduction in the risk of SIDS [26], thus emphasizing the benefits of pacifier use In addition, The American Academy of Pediatric Dentistry (AAPD), however, urges health care providers to follow international guidelines to educate parents about the advantages, risks and safe use of pacifiers, so as to promote healthy infant-children growth and development. Although the above benefits are already documented, the use of pacifiers to support NNS is not welcome in Baby-Friendly Hospitals or those working towards the establishment of Baby-Friendly Hospitals. However, some Baby-Friendly hospitals, following the recommendations of the WHO [2] and UNICEF [27], are encouraged to limit access to pacifiers after childbirth as part of the “Ten Steps” to Successful Breastfeeding” [28]. The Ten Steps summarize a package of policies and procedures that hospitals providing maternity services should implement [2]. Given that the first days of a neonate’s life are not only critical for the neonate, but are an important point of support for mothers to breastfeed successfully, there was the initiative to develop the “10 steps”, which as it was found significantly improve breastfeeding rates [29]. 

Nevertheless, the relationship between pacifier exposure and breastfeeding exclusivity and duration has not been fully elucidated and confusion is createdbecause, the evidence linking pacifiers during neonatal care to subsequent breastfeeding difficulties was limited, consisting mainly of observational studies. So far, there have been several studies that have shown a negative correlation between pacifier exposure and ongoing exclusive breastfeeding [20,30,31], while other studies showed that limiting the distribution of the pacifier to breastfed infants resulted in a reduction in exclusive breastfeeding [32,33,34]. So, pacifier counseling should weigh between the potential protective effects and the potential adverse effects on breastfeeding in preterm infants exiting the NICU. Until now, despite the satisfactory number of reviews on pacifier use and breastfeeding [20,33,34,35], the global literature lacks a systematic review regarding pacifier use in premature infants and the subsequent exclusivity and duration of breastfeeding. Therefore, in order to provide evidence to protect and promote breastfeeding, but also support clinical practices in preterm neonates in NICU, this study aims to perform a systematic review to investigate the relationship between pacifier use in preterm neonates and breastfeeding in infancy.

## 2. Materials and Methods

This systematic review was registered on the PROSPERO prior to starting the investigation (CRD42022352280).

### 2.1. Inclusion and Exclusion Criteria

We included prospective studies, as well as randomized controlled studies that evaluated the association between pacifier use by preterm neonates and breastfeeding in infancy.

Our systematic review followed the guidance of the Preferred Reporting Items for Systematic Reviews and Meta-Analyses (PRISMA) [36,37]. We excluded studies that: (a) were not quantitative including review articles (systematic or not) and letters to the editor; (b) included full-term infants or infants that were not introduced in NICU; (c) combined pacifiers and bottle nipples in the same category; (d) did not report a statistical parameter documenting the size of the association between pacifier use by preterm neonate and duration/exclusivity of breastfeeding. 

### 2.2. Exposure/Intervention: Pacifier Use by Preterm Neonates

The key exposure was pacifier use by preterm neonates in NICU defined as use by preterm neonates in NICU. 

### 2.3. Outcomes: Interruption of Exclusive Breastfeeding

We combined all studies that provided information about pacifier use in preterm neonates and the interruption of exclusive breastfeeding without any further age restrictions. Exclusive breastfeeding was defined as the infant receiving only breast milk and nothing else (allowing oral solutions, syrups, drops and vitamins), according to WHO recommendations [38]. This definition of breastfeeding includes both direct breastfeeding and feeding by pumping breast milk. 

### 2.4. Search Strategy

We searched published articles with the following databases: PubMed/Medline, Google Scholar and Crossref, with language restrictions (only English papers) from March to June 2022.

The search terms used were: preterm neonates OR newborns AND breastfeeding; Pacifier use from preterm neonates OR newborns AND breastfeeding exclusively; Pacifier use from preterm neonates OR newborns AND feeding problems OR feeding difficulties; Pacifier use from preterm neonates OR newborns AND breastfeeding in infancy. 

### 2.5. Study Selection

Three authors (E.A., M.T. and E.O.) evaluated the titles and abstracts independently. Then, the full texts of all potentially relevant papers were retrieved and assessed for eligibility using the predefined inclusion and exclusion criteria defined above. There were no disagreements, hence the consultation of a fourth author. 

### 2.6. Quality Assessment of the Articles

The risk of bias was assessed with a modified version of the Effective Public Health Practice Project Quality Assessment Tool (EPHPP) [39] (Table 1). The six criteria were classified as strong, moderate or weak: (a) selection of bias; (b) study design; (c) confusing factors; (d) blindness; (e) data collection methods and (f) withdrawals and abandonments. Cohort studies and randomized had higher score than cross-sectional studies due to the inability to determine the exposure to the agent and the result. Articles were ranked according to the final rating as strong if none of the quality items were weak; moderate if one of the six items was classified as weak; and weak, for studies with more than one item identified as such [39].

## 3. Results

**Figure 1:**
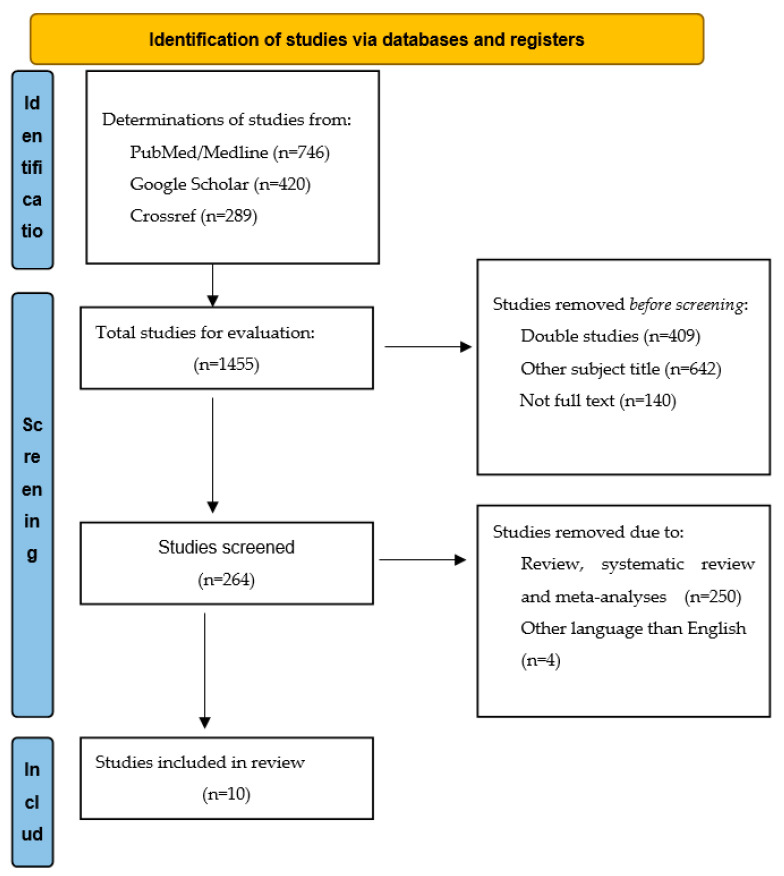
Flow diagram: structure search strategy.

We found 1455 papers in the databases PubMed/Medline, Google Scholar and Crossref. After manual screening of the titles and abstracts of the 1455 studies, excluded 1141 studies. Therefore, a total of 264 studies included for further evaluation. After next screening 253 studies excluded and only 11 articles included in the systematic review (Figure 1). Nine articles were identified as having a strong quality and 2 moderate qualities. The examined item with the more weaknesses was the blinding and the item that had the more moderate was the selection bias (Table 1).

Table 2 shows the included studies characteristics. Most of the articles were performed in Denmark (by the same researcher), followed by Brazil and Turkey. The analysis of the articles was done at two levels: (a) articles showing a positive correlation of the pacifier with breastfeeding and (b) articles showing a negative correlation in the same variables. Most studies were prospective studies or randomized controlled. However, at this point it is worth noting that there was not a specific infant’s age at which the breastfeeding was measured (during the hospitalization, discharge from the hospital, at 1st, 2nd, 4th, 6th month and 12 months to 5 years of age), andfinally, all studies confirmed in-hospital pacifier use in preterm neonates. The gestational weeks of neonates in all surveys ranged from 24 to 36 weeks. In randomized controlled maternal and neonatal characteristics of the groups were similar.

In more details, a Danish prospective study of Maastrup [40] assessing pacifier use during hospitalization in NICU and breastfeeding establishment and exclusivity among 1488 infants between 24–36 gestational weeks. Almost 79% of neonates had their first full oral feeding with breast milk, while breastfeeding was initiated by 21% of extremely premature infants before 30 weekspostmenstrual age. The study however, found a negative effect of pacifier use on breastfeeding in the 1st, 4th, 6th and 12th months of infant’s life. Another prospective study performed by the same researcher [41] also found a negative association between pacifier use and breastfeeding at the same time periods in 1205 preterm infants between 24–36 weeks of gestation. Nevertheless, both studies found additional factors that do not favor the early initiation or exclusive breastfeeding, such as low gestational age, multiple birth, mechanically ventilated infant, primiparity, initiating breast milk expression later than 24 h after delivery and the use of nipple shields. In the Dadalto prospective study [42], 52 mothers of preterm neonates between 30 and 34 weeks participated in the study. This study does not concern pacifier use in the NICU, but after discharge. The attempt to introduce a pacifier took place for 96.2% of preterm infants discharged from NICU. The results, however, show a negative relationship between pacifier use and breastfeeding exclusivity, as well as primiparity, higher hospital length and higher length of orogastric tube. In addition, the retrospective cross-sectional study of Carcavalli published in 2018 [43], investigated the relationship of pacifier use between 250 children (125 preterm and 125full-term). The results of this study show that pacifier use was more prevalent among preterm infants and associated with less than six months of breastfeeding and used of formula-feeding. A very important additional factor associated with reduced prevalence of breastfeeding was the low monthly family income. Thus, pacifier use is subject to economic influences in addition to cultural ones. The prevalence of pacifier use in this study may be related to difficulty initiating breastfeeding for preterm infants, making them more vulnerable to bottle feeding. The randomized study of Fucile [45], evaluated the effect of NNS from an emptied breast versus the use of a pacifier on the setting of breastfeeding at hospital discharge. A total of 33 preterm infants born at less than or equal to 34 weeks’ participated in the study. The results showed that a greater number of neonates in the empty breast group acquired exclusively breastfed at hospital discharge compared to those in the pacifier group. In this study, pacifier use was negatively associated with breastfeeding, while empty breast NNS was associated positively. 

The recent intervention study of Maastrup [46] includes 2 groups (420 and 494 of mother-infant dyads) between 31 to 33 weeks, in order to investigate the educational support breastfeeding program for neonatal nurses through 6 clinical practices that supported breastfeeding. The results of this study show that the infants of the intervention group (reduced pacifier use) had higher rates of exclusive breastfeeding after discharge from the NICU to home, than in the control group. However, exclusive breastfeeding rates in preterm infants at discharge improved after training neonatal nurses in six breastfeeding support clinical practices.

By contrast, some research has shown a positive association of pacifiers with breastfeeding. For explain, the longitudinal study of Kamhawy, published in 2014 [13], randomized 47 preterm infants between 30–34 weeks in NICU to intervention (using pacifier) or to control group (avoid pacifier) during breastfeeding transition. The intervention group showed an accelerated transition to nipple feeding and had better weight gain and earlier discharge than infants without using a pacifier. In this study as well, the infants of the pacifier group apart from the positive results in breastfeeding, they showed higher oxygen saturation, had faster weight gain, and were discharged faster. As it appeared, NNS was found to improve the total physiological and behavioral responses of preterm infants. In addition, the study of Kaya et al. [47] reached similar results to the previous one. In particular, this study randomized 2 groups of preterm infants between 30–34 weeks (pacifier group, n = 34 and control group, n = 36), in order to clarify which of the 2 groups will transition to breastfeeding faster. The results show that infants’ sucking skills in the pacifier group 2 days after switching to oral feeding and before discharge were better than in the control group. Therefore, the use of pacifier improved the sucking skills and reduced the transition time to full breastfeeding and discharge from the NICU. 

The recent randomized study of Say [44] investigated the relationship between the effect of pacifier in preterm infants during the transition to oral feeding, the time to weaning and also the time to full breastfeeding. For the purpose of the study, ninety infants between 26–32 weeks, were randomized into two groups (a pacifier group n = 45 and a control group n = 45).It was observed that the time to transition to full oral feeding, time to transition to full breastfeeding, and time to hospital discharge in the pacifier use group were significantly shorter compared to the control group. Finally, in a more recent single-blind randomized controlled clinical trial of Shaki [5], published in 2022, 150 preterm infants with the gestational age of 31 to 33weeksinto 3 groups (non-nutritive sucking on mother’s finger, pacifier use, controlled) participated in the study. However, the results of this study show that infants in the first 2 groups(non-nutritive sucking on mother’s finger and pacifier use), especially the first one, showed to contribute to increased oral feeding behaviors and thus, a positive effect on the transition to exclusively breastfeeding. 

## 4. Discussion

According to the results, we found a negative association between pacifier use and breastfeeding, mainly in the prospective studies [40,41,42,43] which investigated the long-term effect of pacifier use in infancy. On the other hand, all randomized studies [5,13,44,47] evaluated the short-term effect (after hospital discharge), and found a positive association, except two [45,46] which found a negative association. In general, both prospective and randomized studies provide statistically significant evidence for the pacifier-breastfeeding relationship. Randomized studies have failed to demonstrate the long-term effects on breastfeeding duration or exclusivity. For example, based on these short-term results, we cannot be sure whether the intervention or control groups will continue to have the same results long-term. 

One cross-sectional and most prospective studies indicate additional factors in the negative association of the pacifier and breastfeeding, such as low gestational age, multiple birth, mechanically ventilated infant, primiparity, initiating breast milk expression later than 24 h after delivery, the use of nipple shields, higher hospital length and higher length of orogastric tube, and finally, the low monthly family income [40,41,42,43]. These additional factors suggest that pacifier use in preterm infants and breastfeeding is a complex relationship; therefore, pacifier use in preterm neonates has no real causative effect on breastfeeding in infancy based on the randomized studies.

Our results are in accordance with those of corresponding systematic reviews, even though the sample of this study concerns only premature neonates. For example, no adverse relationship between pacifier use and duration or exclusivity of breastfeeding was supported by a published in 2009 systematic review [33]. Nevertheless, the results of a subsequent systematic review published in 2016 [34] also showed that reduced pacifier use did not improve breastfeeding rates, while the most recent published study [48] showed that the use of a pacifier should not be limited to full-term but also to premature infants as it did not appear to prevent the establishment of breastfeeding. 

Our findings also clearly demonstrated the beneficial effects of NNS on the transition from tube to oral feeding, as it had an effect on reducing NICU length of stay [5,13,47]. Therefore, the introduction of pacifiers to preterm infants in the NICU and especially the use of NNS from the empty nipple or mother’s finger, appear to be beneficial [5,46]. According to the above positive properties of the pacifier, in the revised WHO guidelines for Baby-Friendly Hospitals [28], the risks of using a pacifier are not overestimated, but instructions are provided regarding the potential risks of its use. However, during the hospitalization, the decision to use a pacifier or not in neonates should rest solely with the caregiver.

The major strength of this study is that it is the first to investigate the use of pacifiers exclusively in preterm neonates. The limitations of our results mainly concern the small sample sizes of neonates. Furthermore, there was also some heterogeneity in the interventions of the randomized studies, such as the different ages of the participants, and, not all studies investigated at additional risk factors. None of the included studies examined pacifier use at bedtime alone, which would have assessed the impact of the American Academy of Pediatrics’ recommendation that pacifier be offered at bedtime to reduce the risk of SIDS [26,49]. In future research it would be good to examine the use of a pacifier at bedtime and its effect on breastfeeding.

## 5. Conclusions

According to the results of the systematic review, pacifier use in preterm infants helps transition from tube to oral feeding, breastfeeding, faster weight gain and earlier discharge from the NICU. However, these results are short-term. On a long-term level, the relationship between pacifiers and breastfeeding is more complicated, as it appears to be influenced by additional risk factors. Therefore, further studies focusing on factors that improve breastfeeding rates in preterm infants are necessary both during hospitalization and after discharge from the NICU.

## Figures and Tables

**Table 1:** Methodological Quality of the Studies according to Quality Assessment Tool for Quantitative Studies [39].

Author/Year	Selection Bias	Study Design	Confounders	Blinding	Data Collection Methods	Withdrawals and Drops Out	Final Score
1. Maastrup (2014) [40]	Strong	Moderate	Moderate	Weak	Strong	Strong	Moderate
2. Maastrup (2014) [41]	Strong	Moderate	Moderate	Weak	Strong	Strong	Moderate
3. Dadalto (2016) [42]	Moderate	Moderate	Strong	Strong	Strong	Strong	Strong
4. Carcavalli (2018) [43]	Moderate	Strong	Strong	Moderate	Strong	Strong	Strong
5. Kamhawy (2014) [13]	Moderate	Strong	Strong	Strong	Strong	Strong	Strong
7. Say (2018) [44]	Moderate	Strong	Moderate	Strong	Strong	Strong	Strong
8. Fucile (2021) [45]	Moderate	Strong	Strong	Strong	Strong	Strong	Strong
9. Maastrup (2021) [46]	Strong	Strong	Strong	Strong	Strong	Strong	Strong
10. Shaki (2022) [5]	Moderate	Strong	Strong	Moderate	Strong	Strong	Strong

Notes: All parameters are rated as Strong, Moderate or Weak (receiving 1, 2 or 3 points, respectively) according to specific questions. Section of bias: Are the individuals selected to participate in the study likely to be representative of the target population? (b) What percentage of selected individuals agreed to participate? Study design: (a) the study design; (b) was the study described as randomized? Confounders: (a) Were there important differences between groups prior to the intervention? (b) If yes, indicate the percentage of relevant confounders that were controlled (either in the design (e.g., stratification, matching) or analysis)? Blinding: (a) was (were) the outcome assessor(s) aware of the intervention or exposure status of participants? (b) Were the study participants aware of the research question? Data collection methods: (a) were data collection tools shown to be valid? (b) were data collection tools shown to be reliable? Withdrawals and drops out: (a) Were withdrawals and drop-outs reported in terms of numbers and/or reasons per group? (b) Indicate the percentage of participants completing the study.

**Table 2:** The characteristics of the studies included in the systematic review.

Author/ Country	Design	N	Data	Exposure	Outcome	Outcome Age	Effect of Pacifier Use	Additional Factors
1. Maastrup (2014) [40] Denmark	Prospective	1221 mothers and their 1.488 preterm infants	A national Danish cohort of preterm infants	Pacifier use during breastfeeding transition from neonates 24–36 gestational weeks who were admitted to a NICU	Minimizing the use of a pacifier during breastfeeding transition were associated with earlier establishment of exclusive breastfeeding	1, 4, 6 and 12 months of chronological and corrected age.	Negative	Low gestational age, multiple birth, mechanically ventilated infant, primiparity, initiating breast milk expression later than 24 h after delivery
2. Maastrup(2014) [41] Denmark	Prospective	1205 preterm infants	A national Danish cohort of preterm infants	Pacifier use during breastfeeding transition from preterm neonates with a gestational age of 24–36 weeks	Pacifier use was associated with failure of exclusive breastfeeding	1, 4, 6 and 12 months of chronological and corrected age	Negative	Nipple shields, Delayed initiation of breast milk expression
4. Carcavalli (2018) Brazil [43]	Retrospective comparative Cross-sectional	250 children into two groups	Public hospital and public day-care centre in Belo Horizonte, southwest Brazil	Pacifier use in preterm and full-term infants	Pacifier use was more prevalent among preterm infants and was associated with less than six months of breastfeeding and use of bottle	3 to 5 years	Negative	Low monthly family income
7. Say (2018) [44] Turkey	Prospective, randomized controlled trial	90 Infants in 2 groups (pacifier and control) < 32 weeks	NICU University of Health Sciences Zekai Tahir Burak	Pacifier use in preterm infants up to switching to full breastfeeding	The time for transition to full breastfeeding was shorter in pacifier group infants	Hospital discharge	Positive	
8. Fucile (2021) [45] Canada	Randomized	33 preterm infants < 34 weeks	NICU at Kingston Health Science Centre	Pacifier use or emptied breast from mothers in NICU	Infants in the group of emptied breast sucking acquired exclusive breastfeeds at hospital discharge as compared with those in the pacifier group	Hospital discharge	Negative	
9. Maastrup(2021) [46] Denmark	Intervention study	420 and 494 preterm mother-infant dyads in training program	17 Danish NICUs and one children’s department	Minimizing use of pacifiers	Exclusive breastfeeding rates at discharge from the NICU to home	Hospital discharge	Negative	
10. Shaki (2022) [5] Iran	Single-blind randomized controlled clinical trial	150 preterm infants with the gestational age of 31 to 33 weeks	NICU in Babol Rouhani Hospital, Iran	Finger use or pacifier use or nothing	Breastfeeding Behavior Scale score	Day 10 of interventions	Positive	

## Data Availability

Not applicable.
